# Prognostic significance of β2-microglobulin decline index in multiple myeloma

**DOI:** 10.3389/fonc.2024.1322680

**Published:** 2024-03-18

**Authors:** Tianyu Zhang, Zhili Lin, Ziwei Zheng, Quanqiang Wang, Shujuan Zhou, Bingxin Zhang, Dong Zheng, Zixing Chen, Sisi Zheng, Yu Zhang, Xuanru Lin, Rujiao Dong, Jingjing Chen, Honglan Qian, Xudong Hu, Yan Zhuang, Qianying Zhang, Zhouxiang Jin, Songfu Jiang, Yongyong Ma

**Affiliations:** ^1^ Department of Hematology, The First Affiliated Hospital of Wenzhou Medical University, Wenzhou, Zhejiang, China; ^2^ Department of Neurology, The First People’s Hospital of Fuyang District, Hangzhou, Zhejiang, China; ^3^ Department of Hepatobiliary Surgery, The Second Affiliated Hospital and Yuying Children’s Hospital of Wenzhou Medical University, Wenzhou, Zhejiang, China; ^4^ The First Affiliated Hospital of Wenzhou Medical University, Key Laboratory of Intelligent Treatment and Life Support for Critical Diseases of Zhejiang Province, Wenzhou, Zhejiang, China; ^5^ The First Affiliated Hospital of Wenzhou Medical University, Zhejiang Engineering Research Center for Hospital Emergency and Process Digitization, Wenzhou, Zhejiang, China

**Keywords:** multiple myeloma, β2-microglobulin descending index, β2-microglobulin, prognostic model, the revised international staging system

## Abstract

**Purpose:**

To assess the prognostic significance of β2-microglobulin decline index (β2M DI) in multiple myeloma (MM).

**Methods:**

150 MM patients diagnosed with MM were enrolled in this study. Cox proportional hazards model was used to analyze the uni- and multivariate prognosis in training cohort (n=105). A new combined prognostic model containing β2M DI was built up based on the data in training cohort. The validation group was used to verify the model.

**Results:**

β2M DI showed significant correlation with prognosis in both uni- and multivariate analyses and had a good correlation with complete response (CR) rate and deep remission rate. The ROC and calibration curves in validation cohort (n=45) indicated a good predictive performance of the new model. Based on the median risk score of the training group, we classified patients into high- and low- risk groups. In both training and validation groups, patients in the low-risk group had longer overall survival (OS) time than that in the high-risk group (p<0.05).

**Conclusion:**

β2M DI is a good predictive index for predicting treatment response and survival time in MM patients. The prognostic model added with β2M DI showed a better correlation with OS.

## 1 Introduction

Multiple myeloma (MM) is a malignant monoclonal plasma cell tumor, accounting for about 10% of hematologic tumors which was characterized with anemia, recurrent infection, renal failure, hypercalcemia, ostalgia and pathological fractures (1). The variability of prognostic outcomes for patients with MM is due to the biological and genetic characteristics of myeloma cells and individual factors of the host (2). Therefore, understanding the clinical and host factors associated with prognosis is critical to identifying high-risk populations and individualizing treatment.

The prognostic factors of multiple myeloma mainly include three major aspects: patient factors, tumor characteristics and treatment response. Patient factors refer to the patient’s general condition, including age, fitness status, and physical and mental health (3). Tumor characteristics refer to tumor burden (the number of myeloma cells in the body), the stage of disease progression, and whether cytogenetics are abnormal. The current commonly used Durie-Salmon (DS) stage reflects tumor burden and the Revised International Staging System (R-ISS) is used to assess prognosis. We can assess the patient’s tumor burden and disease progression based on biochemical indicators (e.g., hemoglobin, serum calcium, β2-microglobulin(β2M), serum albumin, lactate dehydrogenase(LDH)) to determine the stage of the disease (4). Response to treatment refers to the efficiency of the treatment on the patient, that is, the degree of remission of the disease. At present, the clinical efficacy evaluation is divided into two criteria——the traditional efficacy criteria (disease progression (PD), partial response (PR), verygood partial response(VGPR), complete response (CR), etc.) and minimal residual disease (MRD) efficacy criteria (5), which require mid-treatment bone marrow detection and complex laboratory equipment (such as second-generation flow cytometry, nested PCR amplification combined with NGS deep sequencing method, PET-CT, etc.). It is therefore desirable to find a routine clinical laboratory test in hospital laboratories to assess patients’ response to treatment and predict survival time.

At present, β2M has been shown to be a routine clinical laboratory test in hospital laboratories and a valid independent predictor of survival in patients with MM (6–9). In our study, we developed a β2-microglobulin descending index (β2M DI) formula based on the change in β2M before and after treatment. We randomly divided patients into training and validation groups. Uni- and multivariate Cox regression analysis showed that β2M DI could better reflect the prognosis of MM patients under treatment than pre-treatment β2M alone. β2M DI showed significant correlation with and had a good correlation with CR rate and deep remission rate. Based on the data in the training cohort, we build a new combined predictive model containing β2MDI. In our study, patients in the low-risk group had longer overall survival (OS) time than that in the high-risk group, and the new model was proved to have good predictive performance. In general, both β2M DI and the predictive model containing β2M DI showed good correlation with OS.

## 2 Patients and methods

### 2.1 Research objects

We collected clinical data of MM patients who were first diagnosed in the First Affiliated Hospital of Wenzhou Medical University from January 2015 to December 2020.Inclusion Criteria: 1. Diagnosis met the diagnostic criteria of the “Guidelines for the Diagnosis and Treatment of Multiple Myeloma” revised by IMWG in 2022 and excluded monoclonal gammopathy of undetermined significance (MGUS) and solitary plasmacytoma. 2. Complete clinical indicators that could be used to determine diagnosis and staging. 3. Had received at least 3 courses of induction chemotherapy in our hospital. Exclusion criteria: 1. Combined with other malignant tumors. 2. Lack of complete review indicators that could assess efficacy. According to the inclusion and exclusion criteria above, a total of 250 patients diagnosed with MM during this period were collected, of which 40 patients were excluded for receiving less than 3 courses of chemotherapy in our hospital, 27 patients were excluded because of irregular follow-up, 32 patients were excluded due to insufficient clinical data to assess their condition, and 1 patient was excluded due to combine with other tumors. At last, 150 patients were included in the study.

Pre-treatment clinical indicators, survival time and survival status of patients were collected through access to inpatient data and outpatient records in the hospital case system and telephone follow-up. The last follow-up for all patients was 2022-5-31.

### 2.2 Definition of β2M DI

when initial serum β2M ≥ 3.5mg/L, β2M DI= (initial serum β2M - serum β2M after treatment)/initial serum β2M; When initial serum β2M < 3.5 mg/L, β2M DI = 1. All patients included in the study received three courses of PIs-based chemotherapy.And post-treatment β2M was defined as β2M after 3 courses of chemotherapy.

### 2.3 Analysis of clinical factors affecting β2M DI

“surv_cutpoint” function of “cutoff” package in R software was used to calculate the maximum selection rank statistic to determine the optimal truncation value of β2M DI. Firstly we used multiple logistic regression to analyze the effect of age (<65; ≥65), sex, creatinine(<177umol/L; ≥177umol/L), LDH (≤245 u/L; >245 u/L), albumin (<35 g/L; ≥35g/L),hemoglobin(<100g/L; ≥100 g/L), corrected serum calcium (total serum calcium (mmol/L) -0.025× serum albumin (g/L) + 1.0 (mmol/L) (≤2.65 mmol/L; >2.65 mmol/L), light chain type (κ;λ), subtype, CD56 (negative; positive) on β2M. And binary logistic regression was used to analyze the effects of the above indicators on β2M DI.

### 2.4 Correlation of β2M DI with treatment response

We assessed patients’ treatment response using CR rate and deep remission rate. Deep remission refers to efficacy of VGPR and above. The difference in treatment response between β2M DI>0.63 and ≤0.63 groups was compared, and the chi-square test was used to verify the statistical difference. p<0.05 was considered statistically significant.

### 2.5 Prognostic analysis and model construction

R 4.1.1 software was used to perform an independent prognostic analysis of clinical features. First, the patients were divided randomly into training and validation cohorts at a 7:3 basis. In the training group, Uni- and multivariate Cox regression method was used to analyze the effects of age, sex, hemoglobin, ISS, RISS, DS stage, albumin, neutrophil, lymphocyte, platelet, β2M, LDH, creatinine, total bilirubin(TB), direct bilirubin(DB),light chain type, corrected serum calcium, monoclonal plasma cell ratio, whether autologous transplantation was performed, and β2M DI on the survival of MM patients. And the variables affecting OS and progression free survival (PFS) time were obtained. P<0.05 was statistically significant. “ggsurvplot” function in R was used to plot the K-M survival curve.

Variables that had significant influence on OS from multivariate analysis were included in the prognostic model predicting 2-, 3- and 4-year survival. The validation group was used to verify the model, and the AUC value and calibration curve predicting the survival rate of 2, 3 and 4 years were obtained.

## 3 Results

### 3.1 Patient characteristics

The median age of 150 patients at diagnosis was 63 (32,85), including 79 males and 71 females. A total of 35 patients underwent autologous transplantation. The median survival of all patients was 33.3 months (7.6, 78.8) and the median progression-free survival was 26.18 months (3.2, 78.8). By the end of follow-up, 95 patients were alive and 55 were dead. The 150 patients were divided into training (n=105) and validation cohorts (n=45) in a ratio of 7:3, and there were no significant differences in the clinical features of the two groups (**Supplementary Table S1**).

### 3.2 Clinical indicators affecting β2M and β2M DI

Serum β2M was normal in 60 out of 150 patients(<3.5mg/L). In the remaining 90 patients, after 3 courses of PIs-based chemotherapy, the serum β2M of 85 patients decreased to varying degrees, with a median decline index of 0.55 (0.04, 0.91), while the serum β2M of 5 patients was higher than the initial treatment with a median decline index of -0.49 (-2.07, -0.01).

Before analyzing the factors affecting β2M DI, to exclude some clinical indicators that may interfere by affecting β2M at the initial diagnosis, we first analyzed the influence of each clinical factor on β2M. We found that there were significant differences in LDH (p=0.015), hemoglobin (p<0.001), and corrected serum calcium (p=0.026) between β2M<3.5mg/L and β2M≥5.5mg/L groups. Between β2M<3.5mg/L and β2M 3.5-5.5mg/L, there was a statistically significant difference between LDH (p=0.005) and hemoglobin (p=0.024) only(p<0.05). The distribution of other parameters in the model were shown in (**Supplementary Tables S2, S3**).

The optimal cut-off value of β2M DI was 0.63 by calculating the maximum selection rank statistic (**Supplmentary Figure S1**). According to this cut-off value, β2M DI was divided into high and low groups. The results showed that creatine (p = 0.010), LDH (p= 0.003), and albumin (p=0.001) had distinct effects on β2M DI (p<0.05). When creatinine<177umol/L, LDH≤ 245u/L, and albumin≥35g/L, β2M DI had an advantage. The distribution of other clinical indicators between β2M DI groups were shown in (**Supplementary Table S4**). In summary, patients with high albumin levels and lower creatinine had greater β2M DI overall and were not disturbed by initial β2M.

### 3.3 Independent prognostic analyses reflect the prognostic value of β2M DI

**Figure 1:**
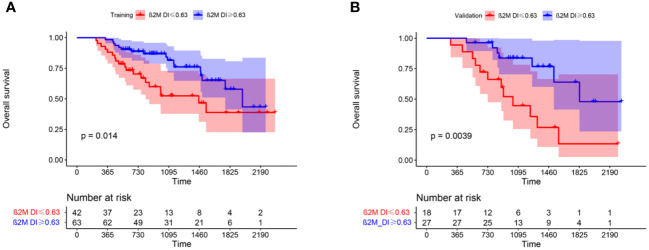
K-M curves showed difference in OS between β2M DI >0.63 and ≤0.63 groups in training **(A)** and validation **(B)** groups.

Univariate analysis showed significant difference in OS between the high and low β2M DI groups in training group(p=0.016). K-M curves in (**Figure 1**) showed that patients in β2M DI>0.63 group had longer OS than those in ≤0.63 group in both training(p=0.014) and validation(p=0.0039) groups. In addition, age, LDH, corrected serum calcium, RISS III, and transplantation were associated with OS for MM in training group(p<0.05); And age, hemoglobin, LDH, creatinine, corrected serum calcium, DS stage III, platelet, and transplantation were showed correlation with PFS in MM patients (p<0.05) (**Table 1**).

**Table 1:** Univariate Cox analysis of factors affecting OS and PFS of MM.

	OS			PFS		
	HR	95% CI	P value	HR	95% CI	P value
Age						
<65	—	—		—	—	
≥65	2.23	1.14, 4.34	0.019	2.16	1.28, 3.62	0.004
Gender						
Female	—	—		—	—	
Male	1.26	0.65, 2.46	0.494	1.11	0.67, 1.84	0.674
Hemoglobin						
<100g/L	—	—		—	—	
≥100g/L	0.59	0.30, 1.16	0.129	0.53	0.32, 0.89	0.016
ISS						
I	—	—		—	—	
II	0.74	0.30, 1.78	0.500	0.69	0.35, 1.35	0.277
III	1.49	0.66, 3.40	0.338	1.26	0.66, 2.38	0.480
RISS						
I	—	—		—	—	
II	1.27	0.51, 3.14	0.604	0.91	0.48, 1.71	0.763
III	3.41	1.17, 9.90	0.024	1.64	0.72, 3.74	0.243
DS stage						
I	—	—		—	—	
II	2.88	0.36, 23.1	0.319	1.64	0.54, 4.94	0.381
III	7.07	0.95, 52.7	0.056	3.17	1.13, 8.91	0.029
Albumin						
<35g/L	—	—		—	—	
≥35g/L	0.82	0.41, 1.62	0.564	0.92	0.55, 1.54	0.748
TB	0.93	0.85, 1.02	0.134	0.96	0.90, 1.03	0.230
DB	0.89	0.72, 1.11	0.315	0.96	0.84, 1.11	0.584
Neutrophil	0.95	0.83, 1.09	0.493	0.87	0.75, 1.01	0.070
lymphocyte	1.00	0.61, 1.64	0.997	0.95	0.65, 1.38	0.774
Platelet	1.00	0.99, 1.00	0.098	0.99	0.99, 1.00	0.001
β2M						
<3.5mg/L	—	—		—	—	
3.5mg/L-5.5mg/L	1.43	0.59, 3.46	0.427	1.12	0.57, 2.21	0.735
≥5.5mg/L	1.68	0.79, 3.54	0.175	1.42	0.81, 2.50	0.217
LDH						
≤245u/L	—	—		—	—	
>245u/L	4.03	1.99, 8.16	<0.001	4.03	1.99, 8.16	<0.001
Creatinine						
<177umol/L	—	—		—	—	
≥177umol/L	4.04	1.80, 9.04	<0.001	2.29	1.17, 4.45	0.015
Corrected serum calcium						
≤2.65mmol/L	—	—		—	—	
>2.65mmol/L	2.52	1.21, 5.27	0.014	1.93	1.09, 3.40	0.024
Light chain type						
κ	—	—		—	—	
λ	1.77	0.92, 3.43	0.090	1.20	0.73, 1.98	0.479
Monoclonal plasma cell ratio	1.01	0.98, 1.03	0.562	1.00	0.98, 1.02	0.983
Transplant						
No	—	—		—	—	
Yes	0.11	0.03, 0.45	0.002`	0.40	0.21, 0.78	0.007
β2M DI						
≤0.63	—	—		—	—	
>0.63	0.45	0.23, 0.86	0.016	0.48	0.26, 0.89	0.020

HR, Hazard Ratio; CI, Confidence Interval.

As albumin, LDH and β2M were collinearity with ISS, RISS, and β2M DI, and hemoglobin, serum creatinine and corrected serum calcium were collinear with DS stage, we excluded these variables in multivariate Cox regression analysis. The results of multivariate analysis showed that ISS (p<0.001(II,III)), RISS (p=0.001(II) and p<0.001(III)), light chain type(p=0.03), transplantation (p=0.004), and β2M DI (p<0.001) were independent prognostic factors of OS (p<0.05); ISS(p=0.012(II) and 0.021(III)), platelet(p=0.006), and β2M DI (p=0.016) were independent factors affecting PFS (p<0.05) (**Table 2**).

**Table 2:** Independent prognostic factors affecting OS and PFS of MM were analyzed by multivariate Cox regression analysis.

	OS			PFS		
	HR	95% CI	P value	HR	95% CI	P value
Age						
<65	—	—		—	—	
≥65	1.28	0.58, 2.83	0.545	1.55	0.81, 2.95	0.181
Gender						
Female	—	—		—	—	
Male	2.11	0.94, 4.70	0.069	1.17	0.68, 2.02	0.568
ISS						
I	—	—		—	—	
II	0.01	0.00, 0.11	<0.001	0.10	0.02, 0.61	0.012
III	0.01	0.00, 0.08	<0.001	0.11	0.02, 0.71	0.021
RISS						
I	—	—		—	—	
II	22.3	3.39, 146	0.001	3.64	0.66, 20.1	0.138
III	75.4	7.24, 785	<0.001	4.11	0.59, 28.4	0.152
DS stage						
I	—	—		—	—	
II	2.23	0.19, 25.6	0.519	1.59	0.48, 5.27	0.446
III	3.76	0.30, 46.7	0.303	2.46	0.76, 7.95	0.131
TB	0.87	0.73, 1.05	0.142	0.94	0.82, 1.07	0.332
DB	1.06	0.77, 1.46	0.721	1.01	0.81, 1.27	0.906
Neutrophil	0.95	0.80, 1.12	0.536	0.88	0.74, 1.04	0.146
lymphocyte	1.34	0.77, 2.32	0.305	1.03	0.68, 1.56	0.876
Platelet	1.00	0.99, 1.00	0.223	0.99	0.99, 1.00	0.006
Light chain type						
κ	—	—		—	—	
λ	2.50	1.09, 5.73	0.030	1.63	0.89, 3.01	0.114
Monoclonal plasma cell ratio	1.01	0.99, 1.04	0.341	0.99	0.97, 1.01	0.280
Transplant						
No	—	—		—	—	
Yes	0.08	0.01, 0.44	0.004	0.50	0.24, 1.07	0.076
β2M DI						
≤0.63	—	—		—	—	
>0.63	0.15	0.05, 0.45	<0.001	0.42	0.21, 0.85	0.016

### 3.4 β2M DI was well correlated with CR rate and deep remission rate after treatment

We assessed the extent of remission in all patients after three courses of chemotherapy. Among the 90 patients with β2M DI>0.63, 23 achieved CR and 24 achieved VGPR. And among the 60 patients with β2M DI ≤ 0.63, the number of people who achieved CR and VGPR was 4 and 15 respectively. The CR rate and deep remission rate of β2M DI>0.63 group were significantly higher than those in β2M DI ≤ 0.63 group (CR rate: 25.6% vs 6.7%, p=0.003; Deep remission rate: 52.2% vs 31.7%, p=0.013) (p<0.05) (**Table 3**).

**Table 3:** Differences in treatment response between β2M DI>0.63 and ≤0.63 groups.

Disease response	β2M DI		P value
	>0.63(n=90)	≤0.63(n=60)	
CR	23(25.6%)	4(6.7%)	0.003
Deep remission (CR+VGPR)	47(52.2%)	19(31.7%)	0.013

### 3.5 β2M DI related prognostic model construction

**Figure 2:**
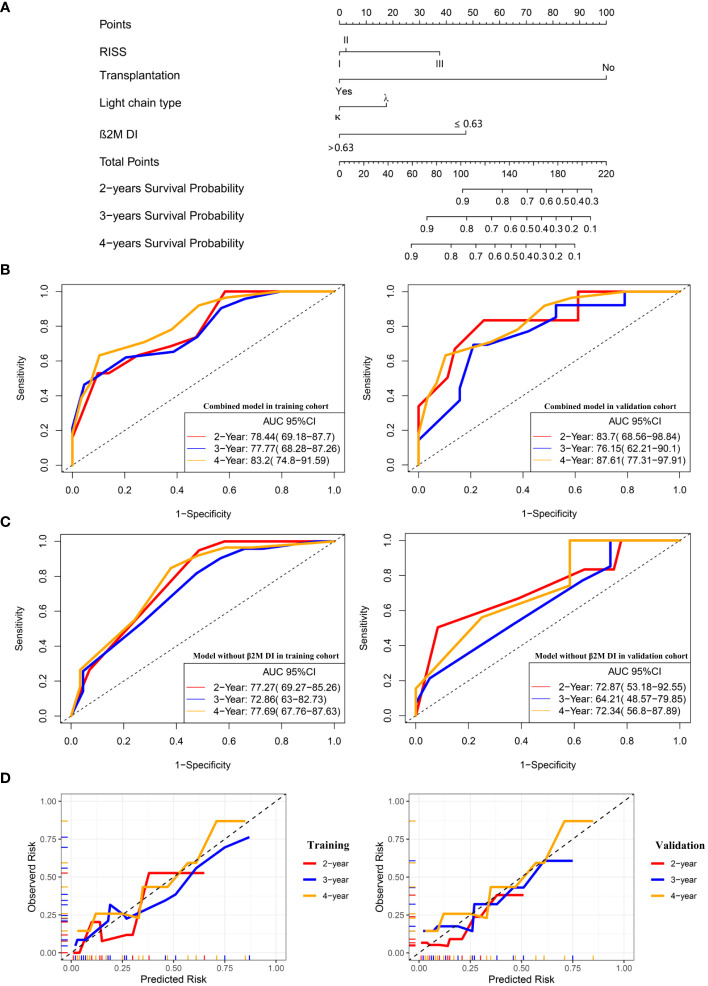
A combined prognostic model was constructed and verified in the validation group. **(A)** The nomogram showed a combined model including RISS, light chain type, transplantation, and β2M DI was built up to predict 2-, 3- and 4-year survivals probabilities. **(B)** ROC curves for the combined model predicting 2-, 3- and 4-year survivals in the training and validation cohort. **(C)** ROC curves for the model without β2M DI predicting 2-, 3- and 4-year survivals in the training and validation cohort. **(D)**The calibration plots of the combined model predicting 2-, 3- and 4-year survivals.

RISS, light chain type, transplantation, and β2M DI were significantly associated with OS in multivariate regression analysis. Therefore, these four variables were used to construct a nomogram predicting 2-, 3- and 4-year survival rates (**Figure 2A**) and calculate the total score for each patient. ROC curve was used to assess the sensitivity and specificity of the combined model. In the training group, the AUC values for 2-, 3- and 4-year survival were 78.44 (95%CI: 69.18, 87.7), 77.77(68.28, 87.26) and 83.2(74.8, 91.59) respectively (**Figure 2B**). In the validation group, AUC values of 2-, 3- and 4-year survival of the predictive model combined with β2M DI increased from 72.87(53.18, 92.55), 64.21(48.57,79.85) and 72.34 (56.8, 87.89) to 83.7 (68.56, 98.84), 76.15(62.21, 90.1) and 87.61(77.31, 97.91) compared with the predicted light chain type, transplantation, and RISS only (**Figures 2B, C**). The calibration plot showed that nomogram containing β2M DI performed well in predicting 2-, 3- and 4-year survivals (**Figure 2D**).

### 3.6 The new combined prognostic model showed good correlation with OS

**Figure 3:**
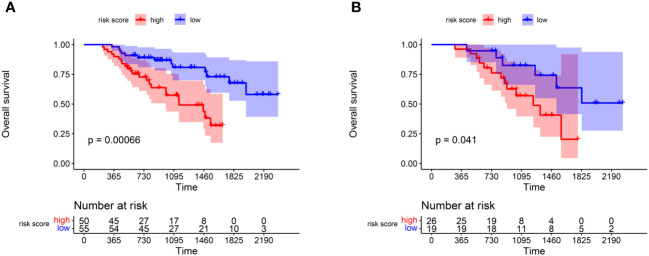
K-M plot reflecting the difference of OS survivals between high and low risk score groups. **(A)** OS comparison of patients in the high and low risk groups in the training group. **(B)** The hazard stratification obtained based on the survival model of the training group was verified in the validation group.

In the training group, we calculated the risk score of each patient according to the newly-built-up prognostic model and divided them into high- and low-risk groups based on the median value. The K-M plot showed that in the training cohort, patients in the low-risk group had longer OS time than those in the high-risk group (p<0.001) (**Figure 3A**). At the same time, we divide the validation group into high- and low-risk cohorts according to the cut-off value in the training group. (**Figure 3B**) showed that patients in the low-risk group in the validation group also had longer survival overall(p=0.041).

## 4 Discussion

β2M is synthesized in all nucleated cells and forms the light chain subunit of the major histocompatibility complex (MHC) class I antigen (10),involving in cell survival, proliferation, and metastasis of various types of cancer (11, 12). Serum β2M levels are directly correlated with tumor burden, as the release of free soluble β2M from the cell membrane or cytoplasm correlates with cell turnover rates (11). At present, serum B2M levels have been extensively studied for their prognostic value in a variety of hematologic disorders. In our study, the β2M DI formula was constructed by comparing the decline rate of β2M after treatment. It was found that β2M DI was an independent prognostic factor in MM patients. The addition of β2M DI to the prognostic model increased the AUC value of the model.

The wide use of PIs has led to a long survival time for most patients, and the β2M level of pHypoproteinemiaatients has continued to change after repeated treatment. Individual differences have led to different treatment responses. The use of pre-treatment β2M alone to assess prognosis has certain limitations. Unlike β2M, β2M DI reflects the decline rate of β2M after treatment, which breaks through the limitations of different patients’ response to plasma cell targeted drugs, so it still showed a good correlation with prognosis in the context of PIs as the main chemotherapy regimen. In our analysis, β2M DI had a good correlation with CR rate and deep remission rate. Traditional efficacy assessment relies on complex methods such as bone marrow testing, while β2M DI only needs to measure the current β2M level at follow-up. We suspect β2M DI could be an indicator that can help clinicians quickly determine a patient’s sensitivity to treatment.

In our analysis, patients with high albumin levels and lower creatinine had greater β2M DI overall and were not disturbed by initial β2M, which suggested that hypoproteinemia and renal insufficiency could slow the decline of β2M. Albumin levels reflect tumor burden and it is a known fact that albumin is associated with the survival of MM (13).We hypothesized that high tumor burden might be one of the cause of lower levels of albumin and poor chemotherapy response. Whether albumin levels reflect chemotherapy response like β2M DI requires further experiment. At the same time, β2M needs to be cleared by the glomeruli. Therefore, patients with renal insufficiency have a slowdown in the decline of β2M due to impaired β2M excretion.

In multivariate regression analysis, MM patients with λ type had a shorter OS than κ type. In a retrospective study, serum free light chains (SFLCs) were significantly higher in patients with monoclonal gammopathy with κ chain lesions than their counterparts with λ chain lesions (14); Free light chains are the cause of renal injury in MM patients (15). Early studies have also shown the role of bortezomib in reducing circulating immune complex and immunoglobulin fragments (16, 17).Therefore, the reason for the longer OS in MM patients with κ chain may be that these patients had lower SFLCs concentrations and faster onset of action for PIs therapy.

In our analysis, autologous stem cell transplantation (ASCT) significantly improved OS in MM patients. Currently, ASCT after high-dose chemotherapy is considered the standard therapy for newly diagnosed MM (18). In the IFM Phase III clinical trial in 2009, the CR rate and minimal residual disease negative rate (p<0.001) in the ASCT group were significantly higher than in the bortezomib-lenalidomide-dexamethasone chemotherapy group alone, which was consistent with the results of our analysis (19).

Historically, therapeutic monitoring of MM has relied primarily on electrophoresis and/or immunofixation to identify and quantify monoclonal proteins in serum and urine samples (5, 20). However, the detection of MRD often requires more complex laboratory equipment (such as second-generation flow cytometry, nested PCR amplification combined with NGS deep sequencing method, PET-CT, etc.). Therefore, we need to develop a simple clinical indicator that can not only avoid the pursuit of demanding CR goals, but also reflect a good response and optimistic survival time.

Retrospective analysis showed that the depth of response was associated with OS and PFS in both patients with medical therapy alone and transplantation (21, 22). β2M represents tumor burden, and its decline often indicates a reduction in tumor burden. Previous report has also shown that β2M was a reliable marker for assessing chemotherapy response and prognosis in patients with MM. Serum β2M and monoclonal proteins were equally predictive in most MM patients (23). Our study confirms that her decline was not only related to the reduction of tumor burden, but also to treatment response and patient survival. The results of the validation group showed that the survival prediction model based on β2M DI improves the AUC value of the traditional model.

Our study also has certain limitations. Firstly, as some patients did not have regular follow-up, we did not analyze the association of β2M DI with the degree of maximum remission. Secondly, The median OS and PFS of patients with MM in this study were shorter than expected, which may be due to the fact that some patients with better underlying conditions were later transferred to outpatient follow-up or treatment in primary hospitals, and these patients were not included in the study due to loss to follow-up, so a multicenter study is needed to confirm whether this conclusion is applicable to the broader population.

In conclusion, β2M DI is a convenient, rapid, and clinically easy to detect method to assess the post-treatment response and long-term prognosis of patients. The addition of β2M DI to the prognostic model significantly improved the sensitivity and specificity of using traditional R-ISS to predict prognosis.

## 5 Conclusion

β2M DI is a good predictive index for predicting treatment response and survival time in MM patients. The prognostic model added with β2M DI showed a better correlation with OS.

## Data availability statement

The raw data supporting the conclusions of this article will be made available by the authors, without undue reservation.

## Ethics statement

The studies involving humans were approved by Ethics Committee in Clinical Research of the First Affiliated Hospital of Wenzhou Medical University. The studies were conducted in accordance with the local legislation and institutional requirements. The participants provided their written informed consent to participate in this study. Written informed consent was obtained from the individual(s) for the publication of any potentially identifiable images or data included in this article.

## Author contributions

TZ: Investigation, Writing – original draft. ZL: Data curation, Investigation, Writing – original draft. ZZ: Investigation, Writing – original draft. QW: Investigation, Writing – original draft. SZ: Investigation, Writing – review & editing. BZ: Investigation, Writing – original draft. DZ: Investigation, Writing – original draft. ZC: Investigation, Writing – original draft. SZ: Investigation, Writing – original draft. YuZ: Supervision, Writing – review & editing. XL: Data curation, Writing – review & editing. RD: Data curation, Investigation, Writing – review & editing. JC: Data curation, Writing – review & editing. HQ: Data curation, Writing – review & editing. XH: Data curation, Writing – review & editing. YaZ: Data curation, Writing – review & editing. QZ: Data curation, Writing – review & editing. ZJ: Supervision, Writing – review & editing. SJ: Supervision, Writing – review & editing. YM: Conceptualization, Funding acquisition, Supervision, Writing – review & editing.

## Supplementary material

Supplementary Figure 1 The optimal cut-off value of β2M DI was obtained by calculating the maximum selection rank statistic.
