# Supplementary figures and images for: Prognostic significance of β2-microglobulin decline index in multiple myeloma
Source: Front Oncol. 2024 Mar 18;14:1322680. doi: 10.3389/fonc.2024.1322680 (PMC10982376; doi:10.3389/fonc.2024.1322680)

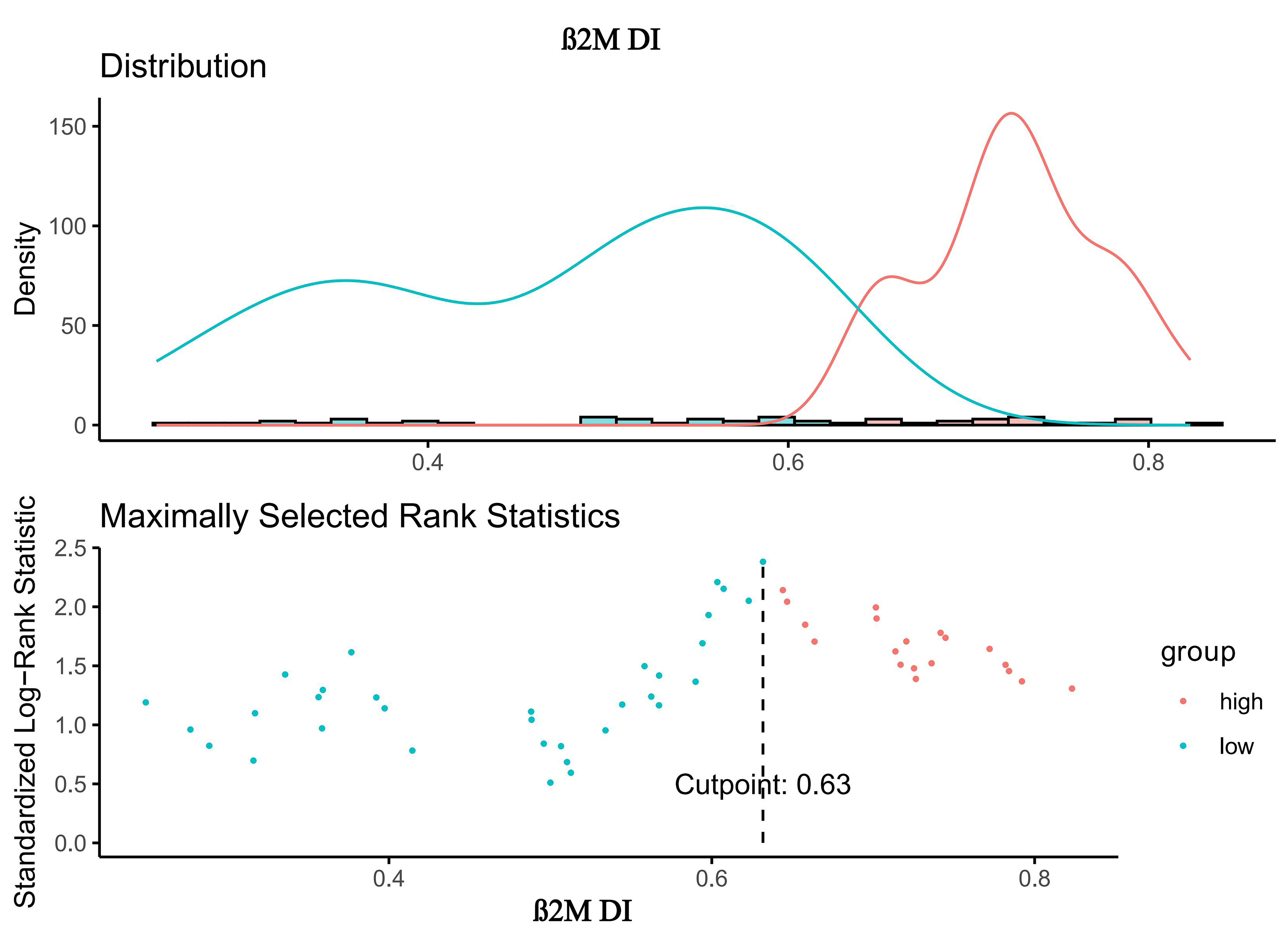

Supplement: Supplementary Figure 1 — The optimal cut-off value of β2M DI was obtained by calculating the maximum selection rank statistic. [file Image_1.jpeg]
