# Supplementary material for: Prognostic significance of β2-microglobulin decline index in multiple myeloma
Source: Front Oncol. 2024 Mar 18;14:1322680. doi: 10.3389/fonc.2024.1322680 (PMC10982376; doi:10.3389/fonc.2024.1322680)
Supplement: Supplementary file 2 [file Table_1.docx]

Table S1 Clinical covariates in the training and validation groups

|  | **Total (n=150)** | **Training（n=105）** | **Validation（n=45）** | **P value** |
| --- | --- | --- | --- | --- |
| Survival status |  |  |  | 0.460 |
| Alive | 95 (63.3%) | 69 (65.7%) | 26 (57.8%) |  |
| Dead | 55 (36.7%) | 36 (34.3%) | 19 (42.2%) |  |
| OS | 999 [658;1475] | 942 [635;1488] | 1031 [780;1462] | 0.618 |
| Disease progression or relapse |  |  |  | 1.000 |
| No | 62 (41.3%) | 43 (41.0%) | 19 (42.2%) |  |
| Yes | 88 (58.7%) | 62 (59.0%) | 26 (57.8%) |  |
| PFS | 786 [508;1158] | 788 [501;1139] | 780 [531;1313] | 0.959 |
| Age |  |  |  | 0.52 |
| <65 | 81 (54.0%) | 59 (56.2%) | 22 (48.9%) |  |
| ≥65 | 69 (46.0%) | 46 (43.8%) | 23 (51.1%) |  |
| Gender |  |  |  | 0.668 |
| Female | 71 (47.3%) | 48 (45.7%) | 23 (51.1%) |  |
| Male | 79 (52.7%) | 57 (54.3%) | 22 (48.9%) |  |
| Hemoglobin |  |  |  | 1 |
| <100g/L | 80 (53.3%) | 56 (53.3%) | 24 (53.3%) |  |
| ≥100g/L | 70 (46.7%) | 49 (46.7%) | 21 (46.7%) |  |
| ISS stage |  |  |  | 0.978 |
| I | 35 (23.3%) | 25 (23.8%) | 10 (22.2%) |  |
| II | 59 (39.3%) | 41 (39.0%) | 18 (40.0%) |  |
| III | 56 (37.3%) | 39 (37.1%) | 17 (37.8%) |  |
| RISS stage |  |  |  | 0.443 |
| I | 28 (18.7%) | 21 (20.0%) | 7 (15.6%) |  |
| II | 100 (66.7%) | 71 (67.6%) | 29 (64.4%) |  |
| III | 22 (14.7%) | 13 (12.4%) | 9 (20.0%) |  |
| DS stage |  |  |  | 0.892 |
| I | 15 (10.0%) | 10 (9.52%) | 5 (11.1%) |  |
| II | 39 (26.0%) | 27 (25.7%) | 12 (26.7%) |  |
| III | 96 (64.0%) | 68 (64.8%) | 28 (62.2%) |  |
| Albumin |  |  |  | 0.072 |
| <35g/L | 85 (56.7%) | 65 (61.9%) | 20 (44.4%) |  |
| ≥35g/L | 65 (43.3%) | 40 (38.1%) | 25 (55.6%) |  |
| Neutrophil | 3.02 [2.25;3.99] | 3.07 [2.23;4.22] | 2.95 [2.32;3.71] | 0.524 |
| lymphocyte | 1.61 [1.16;2.09] | 1.55 [1.13;2.00] | 1.70 [1.35;2.39] | 0.175 |
| Platelet | 188 [134;242] | 188 [126;243] | 188 [148;225] | 0.686 |
| β2M |  |  |  | 0.724 |
| <3.5mg/L | 60 (40.0%) | 44 (41.9%) | 16 (35.6%) |  |
| 3.5mg/L-5.5mg/L | 35 (23.3%) | 23 (21.9%) | 12 (26.7%) |  |
| ≥5.5mg/L | 55 (36.7%) | 38 (36.2%) | 17 (37.8%) |  |
| LDH |  |  |  | 0.824 |
| ≤245u/L | 120 (80.0%) | 83 (79.0%) | 37 (82.2%) |  |
| >245u/L | 30 (20.0%) | 22 (21.0%) | 8 (17.8%) |  |
| TB | 6.50 [2.50;9.00] | 6.00 [2.50;9.00] | 7.00 [2.50;9.00] | 0.918 |
| DB | 3.00 [1.00;4.00] | 3.00 [1.00;4.00] | 3.00 [1.00;3.00] | 0.321 |
| Creatinine |  |  |  | 0.767 |
| <177umol/L | 127 (84.7%) | 90 (85.7%) | 37 (82.2%) |  |
| ≥177umol/L | 23 (15.3%) | 15 (14.3%) | 8 (17.8%) |  |
| Corrected serum calcium |  |  |  | 0.379 |
| ≤2.65mmol/L | 109 (72.7%) | 79 (75.2%) | 30 (66.7%) |  |
| >2.65mmol/L | 41 (27.3%) | 26 (24.8%) | 15 (33.3%) |  |
| Light chain type |  |  |  | 0.372 |
| κ | 80 (53.3%) | 59 (56.2%) | 21 (46.7%) |  |
| λ | 70 (46.7%) | 46 (43.8%) | 24 (53.3%) |  |
| Subtype |  |  |  | 1.000 |
| Light chain | 40 (26.7%) | 28 (26.7%) | 12 (26.7%) |  |
| Heavy chain | 110 (73.3%) | 77 (73.3%) | 33 (73.3%) |  |
| Monoclonal plasma cell ratio | 4.44 [0.92;12.4] | 4.17 [0.84;11.0] | 5.60 [1.70;15.6] | 0.158 |
| Transplant |  |  |  | 1 |
| No | 115 (76.7%) | 80 (76.2%) | 35 (77.8%) |  |
| Yes | 35 (23.3%) | 25 (23.8%) | 10 (22.2%) |  |
| CD56: |  |  |  | 0.239 |
| - | 19 (12.7%) | 16 (15.2%) | 3 (6.67%) |  |
| + | 131 (87.3%) | 89 (84.8%) | 42 (93.3%) |  |
| β2M DI | 0.73 [0.50;1.00] | 0.73 [0.51;1.00] | 0.74 [0.50;1.00] | 0.798 |
