# Supplementary material for: Prognostic significance of β2-microglobulin decline index in multiple myeloma
Source: Front Oncol. 2024 Mar 18;14:1322680. doi: 10.3389/fonc.2024.1322680 (PMC10982376; doi:10.3389/fonc.2024.1322680)
Supplement: Supplementary file 3 [file Table_2.docx]

Table S2 Distribution of clinical parameters between β2M<3.5mg/L and β2M≥5.5mg/L

|  | **β2M** | |  |
| --- | --- | --- | --- |
|  | **＜3.5mg/L(n=60)** | **≥5.5mg/L（n=55）** | **P value** |
| Age |  |  | 0.148 |
| <65 | 36 | 24 |  |
| ≥65 | 24 | 31 |  |
| Gender |  |  | 0.068 |
| Male | 27 | 31 |  |
| Female | 33 | 24 |  |
| Creatinine |  |  | 0.064 |
| <177umol/L | 59 | 37 |  |
| ≥177umol/L | 1 | 18 |  |
| LDH |  |  | 0.015 |
| >245u/L | 4 | 16 |  |
| ≤245u/L | 56 | 39 |  |
| Albumin |  |  | 0.583 |
| <35g/L | 25 | 36 |  |
| ≥35g/L | 35 | 19 |  |
| Hemoglobin |  |  | ＜0.001 |
| <100g/L | 15 | 45 |  |
| ≥100g/L | 45 | 10 |  |
| Corrected serum calcium |  |  | 0.026 |
| >2.65mmol/L | 7 | 28 |  |
| ≤2.65mmol/L | 53 | 27 |  |
| Light chain type |  |  | 0.945 |
| κ | 32 | 26 |  |
| λ | 28 | 29 |  |
| Subtype |  |  | 0.511 |
| Light chain | 17 | 17 |  |
| Heavy chain | 43 | 38 |  |
| CD56 |  |  | 0.509 |
| - | 7 | 6 |  |
| + | 53 | 49 |  |
