# Supplementary material for: Prognostic significance of β2-microglobulin decline index in multiple myeloma
Source: Front Oncol. 2024 Mar 18;14:1322680. doi: 10.3389/fonc.2024.1322680 (PMC10982376; doi:10.3389/fonc.2024.1322680)
Supplement: Supplementary file 4 [file Table_3.docx]

Table S3 Distribution of clinical parameters between β2M<3.5mg/L and β2M 3.5-5.5mg/L

|  | **β2M** | |  |
| --- | --- | --- | --- |
|  | **＜3.5mg/L(n=60)** | **3.5-5.5mg/L(n=35)** | **P value** |
| Age |  |  | 0.673 |
| <65 | 36 | 21 |  |
| ≥65 | 24 | 14 |  |
| Gender |  |  | 0.089 |
| Male | 27 | 21 |  |
| Female | 33 | 14 |  |
| Creatinine |  |  | 0.108 |
| <177umol/L | 59 | 31 |  |
| ≥177umol/L | 1 | 4 |  |
| LDH |  |  | 0.005 |
| >245u/L | 4 | 10 |  |
| ≤245u/L | 56 | 25 |  |
| Albumin |  |  | 0.131 |
| <35g/L | 25 | 24 |  |
| ≥35g/L | 35 | 11 |  |
| Hemoglobin |  |  | 0.024 |
| <100g/L | 15 | 20 |  |
| ≥100g/L | 45 | 15 |  |
| Corrected serum calcium |  |  | 0.687 |
| >2.65mmol/L | 7 | 6 |  |
| ≤2.65mmol/L | 53 | 29 |  |
| Light chain type |  |  | 0.490 |
| κ | 32 | 22 |  |
| λ | 28 | 13 |  |
| Subtype |  |  | 0.471 |
| Light chain | 17 | 6 |  |
| Heavy chain | 43 | 29 |  |
| CD56 |  |  | 0.873 |
| - | 7 | 6 |  |
| + | 53 | 29 |  |
