# Supplementary material for: Prognostic significance of β2-microglobulin decline index in multiple myeloma
Source: Front Oncol. 2024 Mar 18;14:1322680. doi: 10.3389/fonc.2024.1322680 (PMC10982376; doi:10.3389/fonc.2024.1322680)
Supplement: Supplementary file 5 [file Table_4.docx]

Table S4 Effect of clinical indexes on β2M DI in MM patients

|  | **β2M DI** | |  |
| --- | --- | --- | --- |
|  | **＞0.63（n=90）** | **≤0.63（n=60）** | **P value** |
| Age |  |  | 0.948 |
| <65 | 50 | 31 |  |
| ≥65 | 40 | 29 |  |
| Gender |  |  | 0.175 |
| Male | 43 | 36 |  |
| Female | 47 | 24 |  |
| Creatinine |  |  | 0.010 |
| <177umol/L | 82 | 45 |  |
| ≥177umol/L | 8 | 15 |  |
| LDH |  |  | 0.003 |
| >245u/L | 11 | 19 |  |
| ≤245u/L | 79 | 41 |  |
| Albumin |  |  | <0.001 |
| <35g/L | 41 | 44 |  |
| ≥35g/L | 49 | 16 |  |
| Hemoglobin |  |  | 0.214 |
| <100g/L | 39 | 41 |  |
| ≥100g/L | 51 | 19 |  |
| Corrected serum calcium |  |  | 0.067 |
| >2.65mmol/L | 23 | 18 |  |
| ≤2.65mmol/L | 67 | 42 |  |
| Light chain type |  |  | 0.088 |
| κ | 43 | 37 |  |
| λ | 47 | 23 |  |
| Subtype |  |  | 0.996 |
| Light chain | 25 | 15 |  |
| Heavy chain | 65 | 45 |  |
| CD56 |  |  | 0.211 |
| - | 13 | 6 |  |
| + | 77 | 54 |  |
